# Predicting the Properties of High-Performance Epoxy Resin by Machine Learning Using Molecular Dynamics Simulations

**DOI:** 10.3390/nano12142353

**Published:** 2022-07-09

**Authors:** Joohee Choi, Haisu Kang, Ji Hee Lee, Sung Hyun Kwon, Seung Geol Lee

**Affiliations:** 1 School of Chemical Engineering, Pusan National University, Busan 46241, Korea; huenju@pusan.ac.kr (J.C.); jihee9816@pusan.ac.kr (J.H.L.); sunghyun.kwon@pusan.ac.kr (S.H.K.); 2 Department of Chemical and Biomolecular Engineering, University of Illinois at Urbana Champaign, Urbana, IL 61801, USA; haisuk2@illinois.edu; 3 Department of Organic Material Science and Engineering, Pusan National University, Busan 46241, Korea

**Keywords:** epoxy resin, molecular dynamics, machine learning, artificial neural network, adhesive strength

## Abstract

Epoxy resin is an of the most widely used adhesives for various applications owing to its outstanding properties. The performance of epoxy systems varies significantly depending on the composition of the base resin and curing agent. However, there are limitations in exploring numerous formulations of epoxy resins to optimize adhesive properties because of the expense and time-consuming nature of the trial-and-error process. Herein, molecular dynamics (MD) simulations and machine learning (ML) methods were used to overcome these challenges and predict the adhesive properties of epoxy resin. Datasets for diverse epoxy adhesive formulations were constructed by considering the degree of crosslinking, density, free volume, cohesive energy density, modulus, and glass transition temperature. A linear correlation analysis demonstrated that the content of the curing agents, especially dicyandiamide (DICY), had the greatest correlation with the cohesive energy density. Moreover, the content of tetraglycidyl methylene dianiline (TGMDA) had the highest correlation with the modulus, and the content of diglycidyl ether of bisphenol A (DGEBA) had the highest correlation with the glass transition temperature. An optimized artificial neural network (ANN) model was constructed using test sets divided from MD datasets through error and linear regression analyses. The root mean square error (RMSE) and correlation coefficient (*R*^2^) showed the potential of each model in predicting epoxy properties, with high linear correlations (0.835–0.986). This technique can be extended for optimizing the composition of other epoxy resin systems.

## 1. Introduction

Interest in epoxy resins has increased, as they afford the advantages of high bonding and mechanical strength, high chemical resistance and electrical insulation, outstanding dimensional stability, and radiation resistance [1,2]. Epoxy resins are essential in adhesives, coatings, the aerospace industry, electronic materials, fiber-reinforced plastics, and composite materials, and are critical for the next generation of civil engineering materials [3,4,5]. Epoxy adhesives are the most widely used adhesives, and their performance has been verified. These systems are mainly composed of a base resin and a curing agent. Epoxy resins have diverse functional groups with a chemical structure in which the amino groups of the curing agent and epoxy groups of the monomer react with each other. The curing reaction between the epoxy groups in the base resin and the amino groups in the curing agent of typical thermosetting resins creates a crosslinked structure with improved impact, tensile, and bending strengths. Epoxy resins must be designed and modified for use in a variety of applications. An epoxy adhesive system generally requires a certain amount of epoxy monomer and curing agent, the composition of which varies depending on the desired properties. To this end, the use of epoxy resin as an adhesive requires high-level formulation techniques to achieve excellent performance [6,7,8].

In the past decade, effective methods of achieving the desired correlation between the adhesive properties and formulations of epoxy resin have been developed. The types of components differ, and the formulations may vary depending on the component ratio, temperature, and curing conditions, which greatly affect the properties of the epoxy adhesive. Tomonaga et al. [9] used experiments and molecular simulation methods to investigate how differences in the components of an epoxy resin affect the curing properties. Glendimar et al. [10] found a basic relationship between the structure and performance of epoxy resins by investigating three models with high, medium, and low crosslinking densities. However, it is difficult to observe each variation individually because of the large number of formulations. The intricacy of these systems makes quantitative prediction of their basic properties challenging. Therefore, a systematic method for optimizing the formulation of epoxy resins is required. Machine learning (ML), one of the most powerful tools, has been used to solve this problem.

In recent decades, ML techniques have attracted increasing interest in various fields of academia because of their excellent efficiency in extracting information. Recently, ML technology has played a significant role in the design and development of new materials [11]. ML methods can efficiently handle functional corrections in complex epoxy systems, extract information from existing data, and output predicted properties [12,13]. As a data analysis technology, when new data are input, ML generates output values independently from the model established by accumulating training experience from a given dataset, making almost realistic predictions [14,15,16,17]. Clemens et al. [18] improved the components of a composite through ML. Asif et al. [19] studied smart composite laminates using deep learning neural networks. In material science, the primary goal of ML is to search for high-functioning materials with properties tailored to the requirements of a particular application. An artificial neural network (ANN) is a machine learning model and is relatively competitive with conventional linear regression and statistical models in terms of utility [20,21,22]. ANN handles information by learning through experiment with suitable learning samples. It makes deductions by identifying patterns and relationships in data [23,24]. A significant advantage of ANNs is their fast speed and accurate processing provided in large parallel implementations through high-level algorithms because of which they have being gaining increasing attention in research [25,26,27,28]. However, hundreds or thousands of combinations are possible for epoxy resins with multiple components, resulting in an experimental overload when the formulation of the epoxy system becomes complicated. ML generally requires a large amount of data to construct an accurate model. The main constraint is that obtaining datasets through experiments is typically time-consuming and expensive [29,30]. Therefore, comprehensive information on epoxy properties is difficult to obtain. In this study, we used molecular dynamics (MD) simulations to collect sufficient data.

MD simulations can fundamentally reflect the differences in the performance of various resin systems, thus significantly enhancing the efficiency of resin screening in comparison with experimental trial-and-error processes [31,32]. ML coupled with the MD method is a fascinating approach for designing or optimizing epoxy resins, where changes in the characteristics of the resin system components can be automatically determined. Diverse epoxy resin formulations with variable main components are prepared herein, and datasets of the adhesive properties of each formulation are obtained through MD simulations. To predict the properties of high-performance epoxy resins based on different formulations, training is conducted quickly and continuously using an ANN as the ML method. The correlation between the parameters is investigated after a statistical analysis of the database. ANN models are constructed and trained with inspected databases, affording good predictability after optimization. The optimized ANN model is validated using linear regression methods and error analysis. Thus, the properties of the epoxy resin are predicted from information regarding the important constituents. The approach presented herein can be extended to the prediction of complicated formulations of epoxy resins with specific performance targets.

## 2. Materials and Methods

### 2.1. Molecular Dynamics Modeling for Formulations

**Figure 1:**
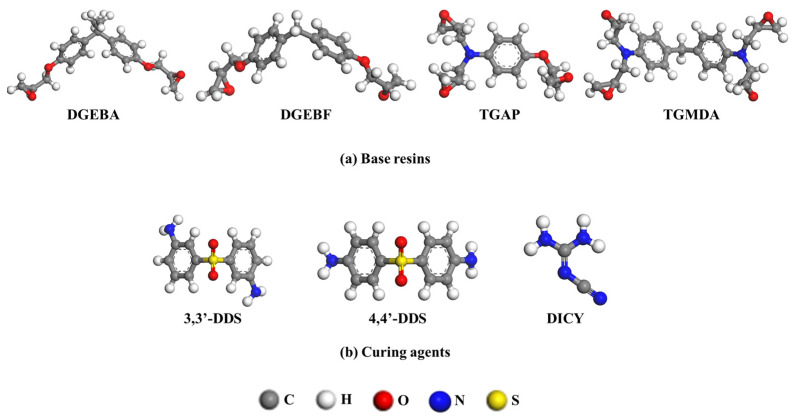
Molecular structures of (**a**) base resins (DGEBA, DGEBF, TGAP, TGMDA) and (**b**) curing agents (33DDS, 44DDS, DICY) that compose epoxy resin systems.

Base resins were diglycidyl ether of bisphenol A (DGEBA), diglycidyl ether of bisphenol F (DGEBF), triglycidyl aminophenol (TGAP), and tetraglycidyl methylene dianiline (TGMDA) (Figure 1a). 3,3-Diaminodiphenyl sulfone (33DDS), 4,4-diaminodiphenyl sulfone (44DDS), and dicyandiamide (DICY) were used as curing agents; the respective structures are shown in Figure 1b. These components have various functional groups, of which the base resins have epoxy groups in common and the curing agents have amine groups in common. In the case of the base resins, all compounds are similar enough that the network structures contain phenylene rings so the epoxy adhesive’s property changes can be compared with relative confidence. DGEBA and DGEBF have two epoxy groups, while TGAP and TGMDA have three and four epoxy groups, respectively. The diamine curing agents also have two amine groups as each amine group can react twice for curing reaction. 33DDS and 44DDS are structurally isomeric diamines, where the substituents on the two phenylene rings are either in meta–meta or para–para arrangement.

All the MD simulations were performed using full-atomistic models. The addition ratio of each base resin and curing agent was determined in accordance with the number of functional groups of each type of epoxy monomer and curing agent. Suitable proportions of the four base resins and the three curing agents were determined. Fifty-four formulations were derived, and the bulk structures corresponding to each formulation were constructed to provide sufficient data for ML. To model the epoxy bulk system, the periodic boundary system (PBC) for the epoxy system was set in the x-y-z direction within the dimensional range of 36.21 Å × 36.21 Å × 36.21 Å to 41.44 Å × 41.44 Å × 41.44 Å according to the formulations.

### 2.2. Simulation Details

**Scheme 1:**
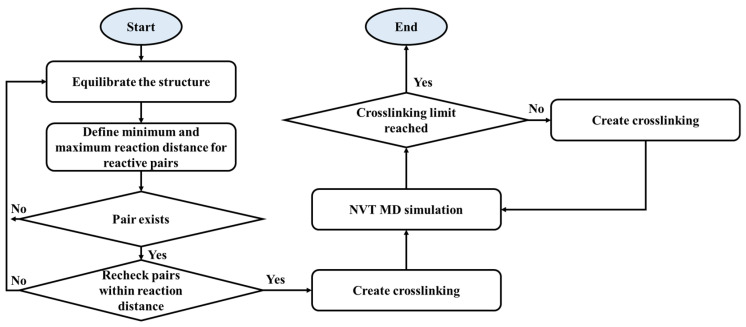
Schematic view of a strategy for crosslinking in MD simulations.

A simulation was performed using Materials Studio software (BIOVIA Software Inc., San Diego, CA, USA), and the polymer consistent force-field (PCFF) was used for the atomic model [33,34]. The potential energy of the molecular structures of the monomer and curing agent was initially minimized using a smart algorithm [35]. The base resin and curing agent were crosslinked through a stepwise mechanism. In the curing reaction, the epoxy groups of the base resins and amine groups of the curing agents react with each other; the ring-opening reaction of the epoxy group leads to the connection of these groups. After the crosslinking reaction, the epoxy group reacts with the primary amine group to form a hydroxyl group. The secondary amine group further reacts with the epoxy group to form a secondary hydroxyl group [36,37]. Other amine groups of the curing agent are crosslinked with other epoxy groups, and other epoxy groups of the monomers connect with other molecules of the curing agent [38]. Each H atom in the amine group participates in the ring-opening reaction. For the curing in MD simulations, the constructed epoxy system model was equilibrated. When the equilibrating step is finished, minimum and maximum reaction distance by 3.5 Å and 10 Å, respectively, were defined for uncrosslinked reactive pairs which indicate carbon of resin’s epoxy group and nitrogen of curing agent’s amine group. If the reaction pair exists within reaction distance, crosslinking is created. As the next step, canonical ensemble (NVT) MD simulation was performed to relax the epoxy structure. This process was repeated until the target crosslinking degree was reached. The degree of crosslinking was set in the 90–100% range. Overall crosslinking reaction scheme is shown in Scheme 1. 

**Figure 2:**
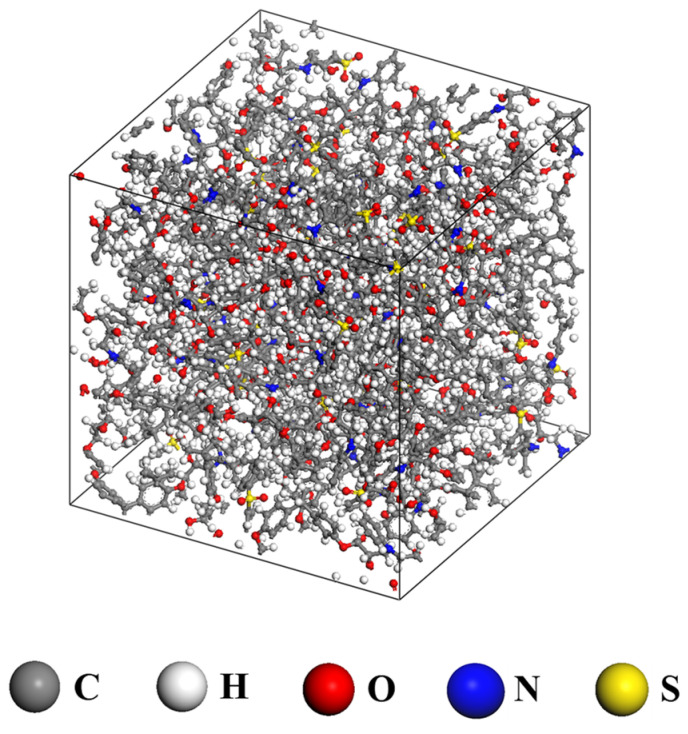
Example of the equilibrated structures of the epoxy resin system.

After building the crosslinked models, NVT MD simulations were performed for 1 ns at 298.15 K for the equilibrium model, and other isothermal–isobaric (NPT) simulations were performed for 1 ns at 298.15 K for geometry optimization (Figure 2). The density was obtained by averaging the values during the last 1 ns of the MD simulations. The other structural analysis, namely, calculating the free volume ratio, was performed using the Connolly surface present within 1.4 Å radius. The free volume was divided by the total volume of the model to compare the relative free volume sizes of the epoxy resin systems.

For the energy analysis of the epoxy resin, the cohesive energy density (CED) of the optimized bulk structure was calculated. The CED is a density that considers only non-bonding energy, such as van der Waals interactions, hydrogen bonds, and Coulomb interactions. The interactions between polymers should be calculated, and as the curing process proceeds, a new bond is formed between various polymers. As one large polymer network is formed, the non-bonding energy between the polymers is reduced; thus, the CED of the corresponding structure is also reduced. Therefore, it is preferable to calculate the CED using the final bulk structure before crosslinking. The CED calculation is given by Equation (1):

(1) $$CED=\frac{E_{cohesive}}{V_{m}}$$

where *E_cohesive_* is the cohesive energy, and *V_m_* is the molar volume of the epoxy system. Stress–strain simulation was performed by applying tensile stress from 0 to 100 MPa to the cured epoxy structure along the loading direction. The strain along the x-, y-, and z-axes can be derived according to the applied tensile load. The modulus was then obtained by dividing the average value of the strain in each direction by the stress. The modulus was determined to confirm the mechanical properties related to deformation of the epoxy adhesives. The glass transition temperature (*T_g_*) is a unique property of an amorphous polymer material that can be used to confirm its thermal characteristics. The annealing process was simulated within the range of 600–300 K. To control the temperature and pressure, the Nose and Berendsen methods were used, respectively [39,40]. The temperature at which the slope of the density graph corresponds to the temperature change is calculated as the glass transition temperature. The input dataset for the ANN model is presented in Table 1. As mentioned above, in this study, numerous epoxy resin systems were built using four types of base resins and three types of curing agents. In addition to being composed of various types of components, a total of 54 formulations were constructed by varying the ratio of the number of molecules added between seven components. In order to achieve ~100% crosslinking degree, the number of molecules of the base resin was used more than the number of molecules of the curing agent. For each epoxy model composed of 54 formulations, a total of 6 properties of epoxy resin systems are calculated by using MD simulations. The degree of crosslinking, density, CED, free volume ratio, glass transition temperature, and modulus in Table 1 are the obtained values.

### 2.3. Machine Learning Modeling

To predict the CED, modulus, and *T_g_* of the epoxy adhesives from simulation datasets, ANN was selected among several ML methods. ANNs are the most effective in predicting and categorizing complicated nonlinear or linear relationships within various parameters [41,42,43,44]. From the dataset for the prediction model, the CED, modulus, and *T_g_* were assigned as the target values, while three independent prediction models were built to achieve a unique target value. Seven compounds (DGEBA, DGEBF, TGAP, TGMDA, 33DDS, 44DDS, and DICY) were used as input values for the independent prediction model. To effectively optimize the ANN model using a low number of input values, each compound was fixed into one type [45]. The values of each parameter in the dataset were based on a number of molecules. Moreover, by altering the ratio of the number of molecules of each compound, the data points were arranged with an appropriate number. Each parameter value was scaled based on the minimum and maximum variables as a standard (min–max normalization) [46].

To establish the initial hyperparameters in terms of the number of hidden layers and neurons of the neural network, 20% of all data points were utilized as selection samples, and 60% of all data points, excluding the selection samples, were categorized as training samples to generalize the primary prediction model. The remaining data points were arranged as test samples to validate their functional capabilities and analyze the ANN model. The steadiest data points with no missing elements were selected as the selection points; however, the training and test samples were randomly separated. The weight and bias were assigned randomly as primary values while training the neural network, and the hyperbolic tangent function (TanH) from the families of sigmoid functions was used as the activation function that controls the weight and bias. The prediction model was trained using the quasi-Newton technique, which approximates the inverse Hassian for every algorithm iteration utilizing gradient information. Numerous ANN prediction models have been optimized using the quasi-Newton technique to achieve accurate predictability [47]. Furthermore, the prediction model was generalized using the quasi-Newton technique with the mean squared error (MSE) settled as a loss function with up to 1000 iterations to optimize the weight and bias variables of the ANN models. MSE determines the mean squared error between the output variables of the ANN model and the target variables of the datasets. The entire procedure was conducted using Neural Designer software [48].

## 3. Results and Discussion

### 3.1. Data Analysis

**Figure 3:**
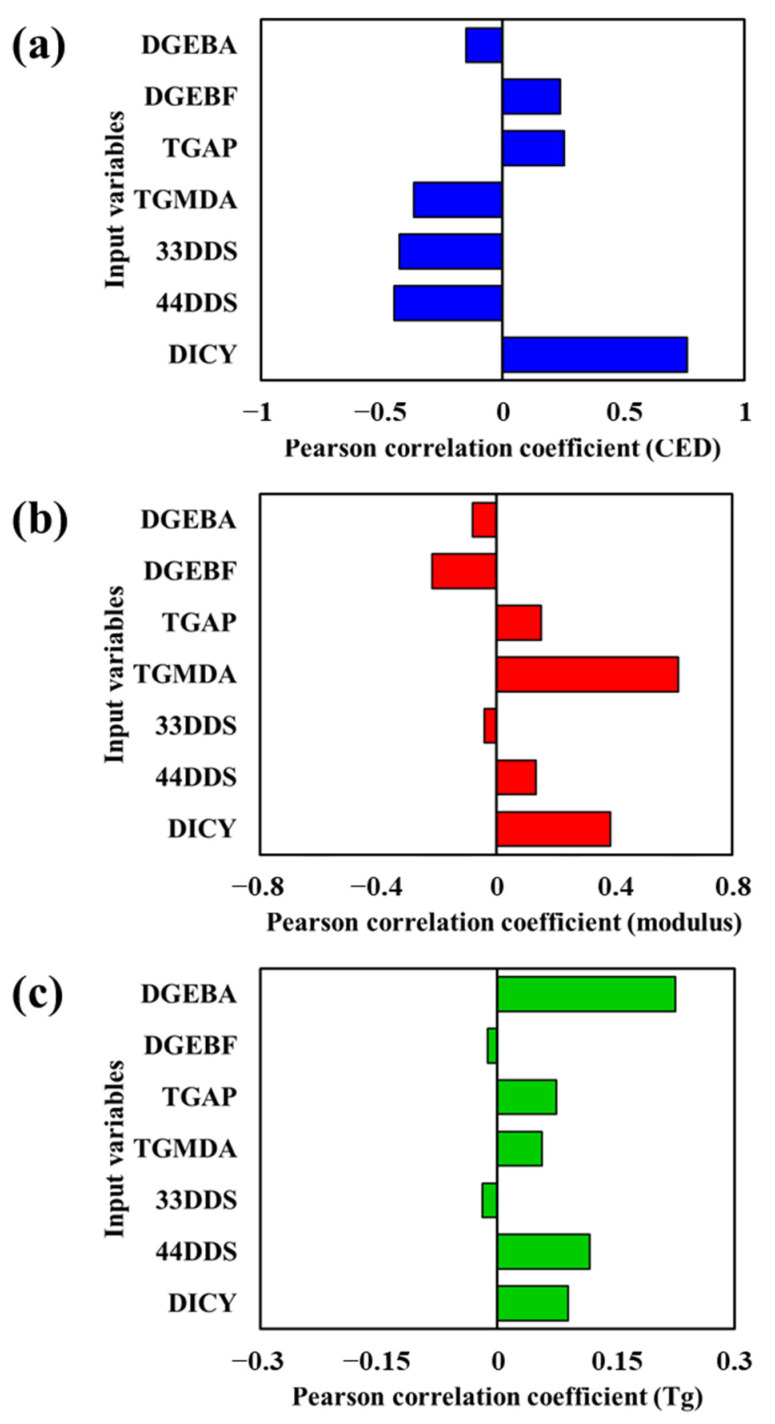
Pearson correlation coefficients for relationships (**a**) between input variables and cohesive energy density, (**b**) between input variables and modulus, and (**c**) between input variables and glass transition temperature.

Before building the ANN model, the datasets acquired from the MD simulations were analyzed (Table 1). A linear correlation analysis was conducted between the input and the target parameters of the dataset to determine the influence of each variable on the CED, modulus, and *T_g_*. The results are shown in Figure 3. The minimum, maximum, and average values of the input variables (including standard deviations) were determined by statistical analysis of the datasets. The linear correlation analysis was performed by measuring the Pearson’s correlation coefficient (PCC). The PCC value was calculated using Equation (2):

(2) $$\mathrm{Pearson}’s\;\mathrm{correlation}\;\mathrm{coefficient}=\frac{n\left(\sum xy\right)-\left(\sum x\right)\left(\sum y\right)}{\sqrt{\left[n\sum x^{2}-{\left(\sum x\right)}^{2}\right]}\;\left[n\sum y^{2}-{\left(\sum y\right)}^{2}\right]}$$

PCC values between −1 and 1 represent the dependence of the target parameters on the input parameters. If the correlation is zero, then the target and input parameters are independent of each other. However, if the PCC has a value of 1 or −1, there is a direct dependence. A value more than 0.5 indicates high correlation, and less than 0.1 indicates low correlation.

Figure 3a shows the PCC for the relationship between the input variable and the CED. The PCC for 33DDS, 44DDS, and DICY were all close to 0.5, and that of DICY exceeded 0.5, showing the highest correlation with the CED. This outcome is an indicator that it is essential to understand the physical properties affecting adhesive strength to prevent cracking when force is applied to high-strength epoxy adhesive. Because the curing reaction of the epoxy resin is not only governed by the curing agent but also by DICY, which has more amine groups than DDS (three amine groups versus two), DICY forms more and stronger bond networks during the curing reaction, thereby validating the observed increase in strength. Therefore, the proportion of the curing agent should be accurately managed. Figure 3b shows the PCC between the input variable and modulus. For TGMDA, the PCC is greater than 0.5, and is the variable having the strongest correlation with the modulus. The highest PCC obtained with TGMDA is reasonable because TGMDA is critical for improving the strength (reflected in the mechanical properties) of the epoxy resin system. Among the base resins that have epoxy groups, TGMDA, with the largest number of epoxy groups (four), undergoes more molecular interactions between the polymer chains and inhibits movement with stronger bonding strength, resulting in higher stiffness. Finally, Figure 3c shows that most of the components, excluding DGEBA, had a PCC close to 0.1. As the reactivity of the polymer network increased, the mobility of the network at a higher temperature increased, which affected thermal characteristics. Therefore, the chemical structure of DGEBA has a strong relationship with the glass transition temperature. The PCC for the effects of DGEBF and TGAP on the CED, modulus, and *T_g_* was found to be less than 0.25. Therefore, the important components strongly affect the CED, modulus, and *T_g_*.

### 3.2. Training Artificial Neural Network

**Figure 4:**
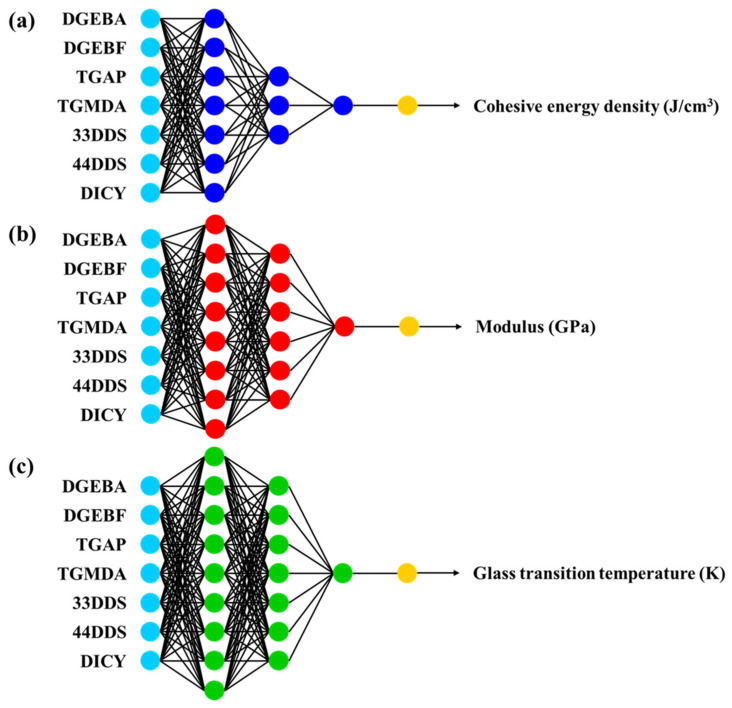
Artificial neural network (ANN) models for predicting (**a**) cohesive energy density, (**b**) modulus, and (**c**) glass transition temperature. The cyan circles of ANN models indicate neurons of input layer, and the blue, red and green circles show neurons of hidden layers. The yellow circles indicate neurons of output layer.

The selection samples from the datasets were generalized by fine-tuning the hyperparameters, and the ANN model was built to obtain the minimum error. The ANN model includes three types of layers (one input layer, three hidden layers, and one output layer (Figure 4). The hyperparameters of the ANN model comprise the hidden layers and neurons: the CED prediction model consists of three hidden layers and seven, three, and one neuron per layer (Figure 4a). For the modulus ANN model, three hidden layers and eight, six, and one neuron(s) per layer were used (Figure 4b). The final *T_g_* prediction model had three hidden layers and nine, seven, and one neuron(s) per layer (Figure 4c). These initial models were designed as training samples with optimization for minimum loss. To predict the CED, modulus, and *T_g_*, the optimized ANN models were implemented by using the training and test samples. Table 2 shows the measured root mean square error (RMSE) and normalized squared error (NSE) for each prediction model.

For the CED ANN model, the training and test samples presented small RMSEs of 1.413 and 1.124, respectively. Considering that the standard deviation of the CED properties in the datasets was 30.138, the developed CED prediction model presents excellent accuracy. For the modulus, the developed prediction model had an RMSE of 0.223 for the training samples and 0.346 for the test samples. The standard deviation of the modulus in the sets was 1.136; thus, the modulus prediction model exhibits good accuracy for the training samples. For the *T_g_* ANN model, the training and test samples presented small RMSEs of 1.811 and 2.581, respectively. The developed ANN model for *T_g_* prediction presented outstanding accuracy compared with the standard deviation of the *T_g_* values in the datasets, which was 39.061.

**Figure 5:**
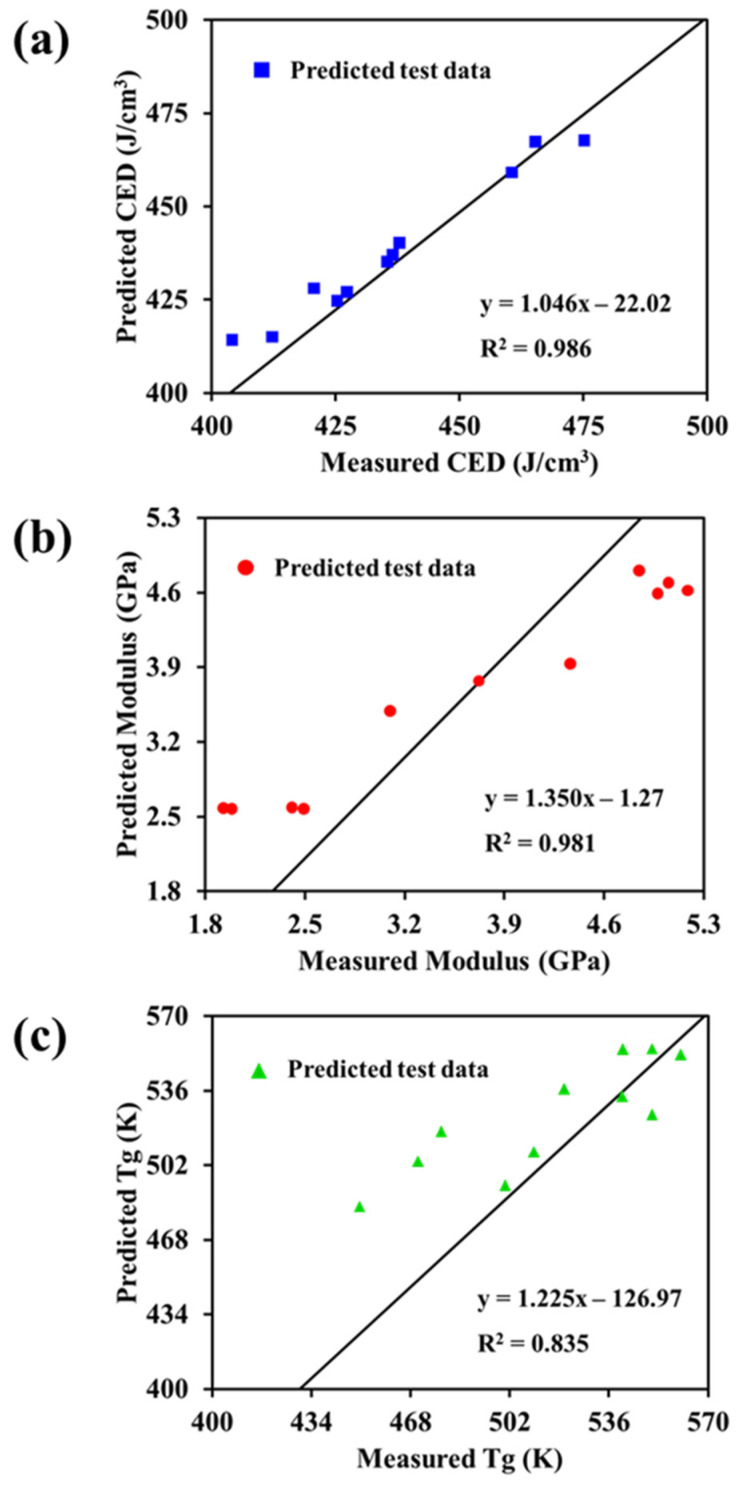
(**a**) Correlation of cohesive energy density predicted by ANN with actual cohesive energy density, (**b**) correlation of modulus predicted by ANN with actual modulus, and (**c**) correlation of glass transition temperature predicted by ANN with actual glass transition temperature.

The ANN model was further verified through linear regression analysis. The results are shown in Figure 5. Linear regression analysis was performed by determining the regression line between the predicted properties and the calculated properties of the datasets. For a perfect correlation between the output data of the prediction model and the target value, the slope of the linear regression should be 1 and the y-intercept should be 0. Figure 5 shows that the data predicted by the CED, modulus, and *T_g_* ANN models presented a linear correlation with the calculated data. The determination coefficients (*R^2^*) were 0.986, 0.981, and 0.835 for the CED, modulus, and *T_g_* ANN models, respectively, indicating that the developed prediction model is highly accurate.

## 4. Conclusions

A method for designing epoxy resins based on MD simulations and an ML method was established. This combining technique has been suggested to predict and optimize the adhesive properties of the epoxy amine system composed of four different base resins and three different curing agents with numerous formulations. The proposed method can be used to determine the effects of various constituents on the adhesive properties of epoxy rapidly, precisely, and automatically. MD simulations were used to construct numerous datasets. Prediction models were constructed and implemented for the CED, modulus, and *T_g_* of epoxy resins with complex formulations using an ANN. A compendium of the adhesive property data (CED, modulus, and *T_g_*) was first prepared for samples with diverse formulations. Each dataset contained seven constituents: DGEBA, DGEBF, TGAP, TGMDA, 33DDS, 44DDS, and DICY.

A data analysis confirmed that among epoxy resin components, the curing agent DICY had the strongest correlation with the CED value. The TGMDA resin formulation showed the greatest correlation with the modulus. However, DGEBA, which is the base resin, showed a strong correlation with *T_g_*. This analysis offers a physical understanding of epoxy adhesives over a broad range of formulations for preparation. Quantitative analysis indicates the relationship between the input values and the target values by using PCC values. The analyzed data were used to construct and optimize ANN models to predict the CED, modulus, and *T_g_* by dividing the data into selection, training, and test sets. In terms of prediction, the optimized ANN model achieved RMSEs of 1.413 and 1.124 for CED, and 0.223 and 0.347 for the modulus on the training and test sets, respectively. The ANN model for *T_g_* achieved RMSE values of 1.811 and 2.581 on the training and test sets, respectively. The ANN models were optimized by linear regression analysis. In the predictions of the CED, modulus, and *T_g_*, the *R*^2^ values were 0.986, 0.981, and 0.835, respectively. The analysis indicates that the optimized ANN model enables reasonable prediction of the CED, modulus, and *T_g_* of epoxy resins. 

From an initial dataset of measured epoxy system properties with different formulations, our learning model was able to build a predictive ANN model of CED, modulus, and Tg with high accuracy. Since our ANN model was constructed with a low number of datasets, and prediction accuracy was significantly high, this approach is expected to reduce the time and cost of material design and development, especially when it is difficult to characterize numerous epoxy system properties by experiments one by one. Future research related to this point should aim to optimize the properties of the epoxy resins according to diverse formulations. The application of this study allows the addition of different composition types of the epoxy adhesives and allows the ratio of the number of added molecules to datasets to increase the design of freedom of high-performance epoxy resins. To achieve future work development, our ANN prediction model is essential to characterize the structural, mechanical and thermal properties of the epoxy adhesive applications, and also to reduce the challenge of constructing an epoxy resin model for complex formulations. Therefore, we expect that our work will offer an innovative pathway for developing high-performance epoxy resins by predicting complicated formulations of epoxy resins to achieve specific target performance.

## Figures and Tables

**Table 1:** Dataset from MD simulations for machine learning.

Sample	DGEBA	DGEBF	TGAP	TGMDA	33DDS	44 DDS	DICY	Crosslinking Degree (%)	Density (g/cm^3^)	Cohesive Energy Density (J/cm^3^)	Free Volume (%)	Glass Transition Temperature (K)	Modulus (GPa)
1	1	0	0	0	0.5	0	0	93	1.146	428.2	0.085	500	2.499
2	1	0	0	0	0	0.5	0	90	1.154	420.6	0.087	569	1.908
3	1	0	0	0	0	0	0.5	93	1.129	460.3	0.071	420	1.820
4	0	1	0	0	0.5	0	0	93	1.198	446.5	0.084	478	2.415
5	0	1	0	0	0	0.5	0	93	1.163	445.8	0.092	531	2.053
6	0	1	0	0	0	0	0.5	91	1.194	490.9	0.066	530	2.235
7	0	0	1	0	0.75	0	0	90	1.228	429.5	0.110	450	3.717
8	0	0	1	0	0	0.75	0	92	1.185	433.6	0.120	550	3.101
9	0	0	1	0	0	0	0.75	93	1.220	506.2	0.106	450	4.522
10	0	0	0	1	1	0	0	93	1.184	400.1	0.108	490	3.785
11	0	0	0	1	0	1	0	92	1.207	398.6	0.105	400	4.347
12	0	0	0	1	0	0	1	91	1.220	453.1	0.088	520	5.221
13	1	1	0	0	1	0	0	93	1.174	436.2	0.034	570	2.151
14	1	1	0	0	0	1	0	93	1.166	439.1	0.033	550	1.969
15	1	1	0	0	0	0	1	93	1.156	476.5	0.028	470	2.024
16	1	0	1	0	1.25	0	0	93	1.173	427.2	0.043	540	2.797
17	1	0	1	0	0	1.25	0	92	1.162	426.4	0.046	520	2.072
18	1	0	1	0	0	0	1.25	91	1.172	482.8	0.029	530	3.271
19	1	0	0	1	1.5	0	0	93	1.169	407.2	0.041	480	3.126
20	1	0	0	1	0	1.5	0	90	1.197	406.9	0.043	570	3.818
21	1	0	0	1	0	0	1.5	93	1.190	459.2	0.032	520	4.403
22	0	1	1	0	1.25	0	0	93	1.203	441.2	0.044	490	3.127
23	0	1	1	0	0	1.25	0	91	1.192	437.9	0.050	560	2.041
24	0	1	1	0	0	0	1.25	93	1.201	503.2	0.035	510	4.514
25	0	1	0	1	1.5	0	0	92	1.191	422.1	0.047	490	3.100
26	0	1	0	1	0	1.5	0	91	1.170	416.9	0.045	510	3.270
27	0	1	0	1	0	0	1.5	91	1.188	477.6	0.032	550	5.079
28	0	0	1	1	1.75	0	0	93	1.199	414.6	0.054	530	4.963
29	0	0	1	1	0	1.75	0	93	1.202	414.3	0.054	540	4.757
30	0	0	1	1	0	0	1.75	91	1.198	479.3	0.044	550	4.454
31	2	0	1	0	1.75	0	0	90	1.182	425.3	0.029	510	2.254
32	2	0	1	0	0	1.75	0	93	1.162	426.6	0.039	570	3.891
33	2	0	1	0	0	0	1.75	93	1.163	475.1	0.032	550	3.382
34	2	0	0	1	2	0	0	92	1.163	412.0	0.039	520	4.876
35	2	0	0	1	0	2	0	93	1.163	413.1	0.038	500	4.410
36	2	0	0	1	0	0	2	92	1.171	454.0	0.032	560	4.356
37	0	2	1	0	1.75	0	0	93	1.176	446.3	0.043	450	2.389
38	0	2	1	0	0	1.75	0	91	1.191	438.7	0.042	490	3.126
39	0	2	1	0	0	0	1.75	92	1.186	497.6	0.034	540	3.514
40	0	2	0	1	2	0	0	93	1.188	425.3	0.036	560	2.295
41	0	2	0	1	0	2	0	93	1.195	421.0	0.035	500	3.783
42	0	2	0	1	0	0	2	93	1.200	484.8	0.029	550	4.411
43	1	0	2	0	2	0	0	93	1.210	427.2	0.043	540	4.178
44	1	0	2	0	0	2	0	93	1.205	427.1	0.042	550	4.510
45	1	0	2	0	0	0	2	91	1.202	490.3	0.029	540	4.858
46	1	0	0	2	2.5	0	0	92	1.188	403.9	0.037	510	5.310
47	1	0	0	2	0	2.5	0	92	1.168	404.4	0.044	550	5.041
48	1	0	0	2	0	0	2.5	92	1.157	458.5	0.039	500	5.321
49	0	1	2	0	2	0	0	93	1.184	434.0	0.049	550	3.256
50	0	1	2	0	0	2	0	93	1.201	435.5	0.051	460	5.167
51	0	1	2	0	0	0	2	93	1.236	504.1	0.043	510	4.876
52	0	1	0	2	2.5	0	0	92	1.172	410.1	0.047	540	4.847
53	0	1	0	2	0	2.5	0	90	1.176	411.0	0.047	540	5.009
54	0	1	0	2	0	0	2.5	93	1.190	465.2	0.037	490	5.311

**Table 2:** Root mean squared error (RMSE) and normalized squared error (NSE) on the training and test sets for predicting the cohesive energy, modulus, and glass transition temperature using the ANN model.

	Cohesive Energy Density		Modulus		Glass Transition Temperature	
	Training Sample	Test Sample	Training Sample	Test Sample	Training Sample	Test Sample
RMSE	1.413	1.124	0.223	0.346	1.811	2.581
NSE	0.153	0.039	0.075	0.103	0.209	0.395

## Data Availability

The data presented in this study are available on request from the corresponding author.
